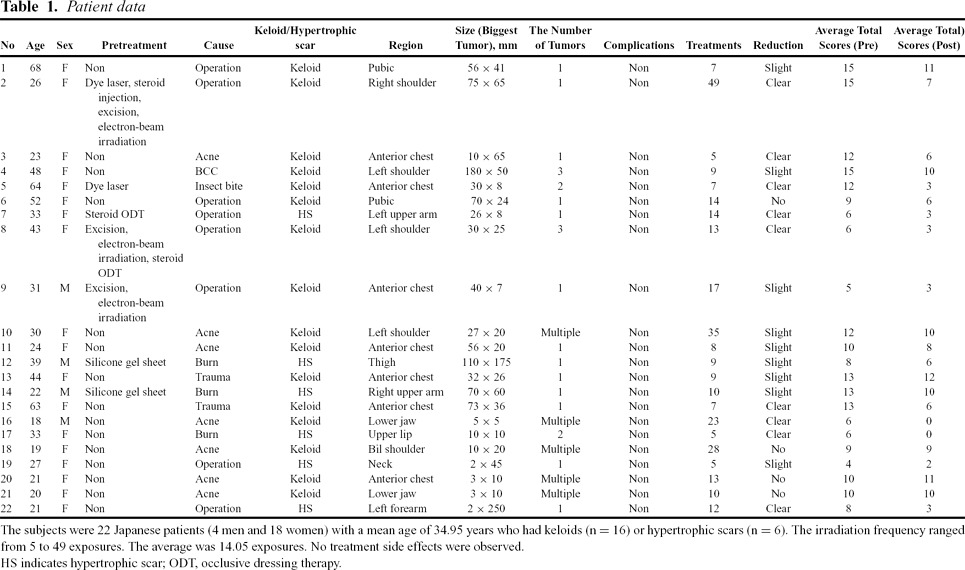# Nd:YAG Laser Treatment of Keloids and Hypertrophic Scars

**Published:** 2012-01-11

**Authors:** Satoshi Akaishi, Sachiko Koike, Teruyuki Dohi, Kyoko Kobe, Hiko Hyakusoku, Rei Ogawa

**Affiliations:** Department of Plastic, Reconstructive and Aesthetic Surgery, Nippon Medical School, Tokyo, Japan

## Abstract

Pathological cutaneous scars such as keloids and hypertrophic scars (HSs) are characterized by a diffuse redness that is caused by the overgrowth of capillary vessels due to chronic inflammation. Our group has been using long-pulsed, 1064-nm Nd:YAG laser in noncontact mode with low fluence and a submillisecond pulse duration to treat keloids and hypertrophic scars since 2006 with satisfactory results. The present study examined the efficacy of this approach in 22 Japanese patients with keloids (n = 16) or hypertrophic scars (n = 6) who were treated every 3 to 4 weeks. Treatment settings were as follows: 5 mm spot size diameter; 14 J/cm^2^ energy density; 300 μs exposure time per pulse; and 10 Hz repetition rate. The responses of the pathological scars to the treatment were assessed by measuring their erythema, hypertrophy, hardness, itching, and pain or tenderness. Moreover, skin samples from 3 volunteer patients were subjected to histological evaluation and 5 patients underwent thermography during therapy. The average total scar assessment score dropped from 9.86 to 6.34. Hematoxylin and eosin staining and Elastica Masson-Goldner staining showed that laser treatment structurally changed the tissue collagen. This influence reached a depth of 0.5 to 1 mm. Electron microscopy revealed plasma protein leakage, proteoglycan particles, and a change in the collagen fiber fascicles. Further analyses revealed that noncontact mode Nd:YAG laser treatment is highly effective for keloids and hypertrophic scars regardless of patient age, the origin and multiplicity of scarring, the location of the scar(s), or the tension on the scar.

Pathological cutaneous scars such as keloids and hypertrophic scars (HSs) are characterized by a diffuse redness that is caused by the overgrowth of capillary vessels because of chronic inflammation.^1^ The pulsed dye laser (PDL) treatment of scars was first described by Alster et al^2^ in 1993, after which it rapidly became a mainstream form of laser treatment of scars. Pulsed dye laser has a high absorption coefficient for hemoglobin and is used to reduce the redness and thickness of cutaneous scars.^3^ Although PDL is more effective than argon and carbon dioxide lasers, which target the water in the skin,^4^^-^7 a single PDL irradiation procedure is not sufficient to completely improve the scar surface conditions. Consequently, to obtain better clinical outcomes, PDL should be combined with corticosteroid injections and/or 5-fluorouracil treatment.^8^^,^^9^

Our group has been using a long-pulsed, 1064 nm Nd:YAG laser in noncontact mode with low fluence and submillisecond pulse duration to treat keloids and HSs since 2006^10^^-^12 with satisfactory results. This paper examines the efficacy, adaptability, and limitations of this treatment. Moreover, to elucidate the mechanism by which this treatment modality acts, biopsies of irradiated and nonirradiated keloids were subjected to histopathological and microscopic analysis.

## STUDY DESIGN AND METHOD

In total, 22 Japanese patients (4 men and 18 women) with a mean age of 34.95 years were enrolled. Of these patients, 16 had keloids and 6 had HSs (Table 1). In this study, a *keloid* was defined as a scar that extended beyond the confines of the original wound. The patients were treated every 3 to 4 weeks with the long-pulsed, 1064 nm Nd:YAG laser (Genesis, Cutera Inc, Brisbane, California), which was applied in noncontact mode.

The laser irradiation was applied in a zigzag fashion by a hand piece held 2 to 3 cm above the skin surface. The treatment settings were as follows: a spot-size diameter of 5 mm, an energy density of 14 J/cm^2^, an exposure time per pulse of 300 μs (0.3 ms), and a repetition rate of 10 Hz (500 to 1000 pulses/cm^2^). The copper cooling tip of the hand piece was not used to cool the skin before or after treatment. To prevent the overheating of the epidermis, the treatment area was cooled by an iced gel pack before, during, and after laser irradiation. A strong (betamethasone butyrate propionate) or very strong steroid (clobetasol propionate) ointment was administered at 0.1 g/cm^2^ for 2 to 3 days after the treatment to reduce the chance of a bulla developing after irradiation. The total number of treatments ranged from 5 to 49 exposures. On average, the patients received 14.05 exposures.

## CLINICAL EVALUATION

The responsiveness of the pathological scars to the treatment was assessed by measuring the following 5 parameters: erythema, hypertrophy, hardness, itching, and pain or tenderness. The assessments were performed by 3 independent experienced plastic surgeons before the treatments started and after they were completed. Scoring was based on a 4-point scale that ranged from 0 to 3 (0 = absent, 1 = mild, 2 = moderate, 3 = severe). Thus, the maximum possible score for a patient was 15 points. The data were analyzed statistically by using the Mann-Whitney *U* test.

## HISTOLOGICAL EVALUATION

Three volunteer patients who had multiple keloids on their chest and were scheduled for keloid excisions provided skin samples for histological evaluation. Thus, for each patient, half of the scar was irradiated by laser 3 days before the excision. In total, 500 to 1000 pulses were delivered per cm^2^. After excision, the histopathology of the specimens was examined by hematoxylin and eosin staining and Elastica Masson-Goldner staining. The tissue specimens were then examined under an electron microscope. These 3 patients were not among the 22 patients who were enrolled in the trial.

## THERMOGRAPHIC EVALUATION

Five patients with keloids agreed to assessments of the temperature of their treated skin surface. Thus, a thermograph (TVS-700, Nippon Avionics Co, Ltd, Tokyo, Japan) was used to measure the skin temperature 5 times during the procedure. Of the 5 patients, 2, 1, and 2 had lesions on their chest, lower abdomen, and shoulder, respectively. Furthermore, fresh ex vivo pigskins were irradiated and analyzed with a thermograph to determine the depth to which the laser heat-inducing influence penetrated. The results were analyzed by using AVIO PE Professional software (version 3.12, Nippon Avionics Co, Ltd, Tokyo, Japan).

## RESULTS

**Figure 1:**
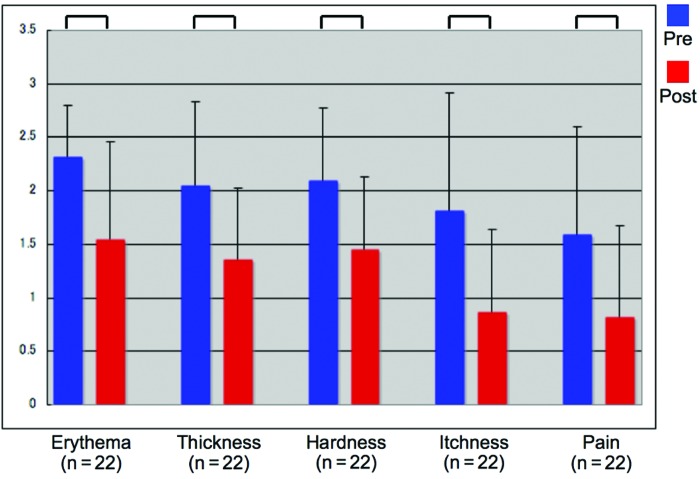
Average assessment scores for all keloid and hypertrophic scars before and after laser treatment. Mann-Whitney *U* tests revealed significant improvements in scar erythema, thickness, hardness, itchiness, and pain (*P* < .01 for all) after an average of 14.05 exposures (range, 5-49). In particular, the subjective symptoms improved after irradiation.

The average total scar assessment score fell from 9.86 to 6.34 after irradiation. Of the 22 patients, 8 exhibited a clear reduction in the size of their lesions (<90% of the original area), 10 had a slight reduction (90% 95%), and 4 showed no change (>95%). The average scores of the 5 scar parameters (erythema, hypertrophy, hardness, itching, and pain or tenderness) before and after the treatments are shown in Figure 1. In particular, there was a remarkable improvement in itchiness scores. Mann-Whitney *U* tests revealed that all the parameters had improved significantly after treatment was completed (*P* < .01 for all comparisons) (Fig 1).

**Figure 2:**
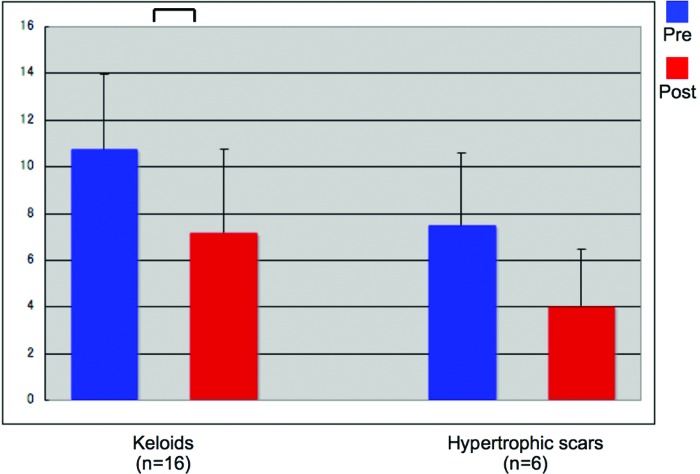
Average total scores of the keloids and hypertrophic scars before and after treatment. The laser treatment effectively improved the keloids, as indicated by Mann-Whitney *U* testing (*P* < .01). In contrast, the treatment was not as effective with hypertrophic scars (*P* = .058). This may reflect the relatively small number of hypertrophic scars that were examined.

The scars were then divided into 2 groups depending on whether they were keloids or HSs and the change in their average total scores after treatment was assessed. The laser treatment effectively improved the keloids, as indicated by the Mann-Whitney *U* tests (*P* < .01). While the HSs also showed some improvement, this did not achieve statistical significance. (*P* = .058) (Fig 2).

**Figure 3:**
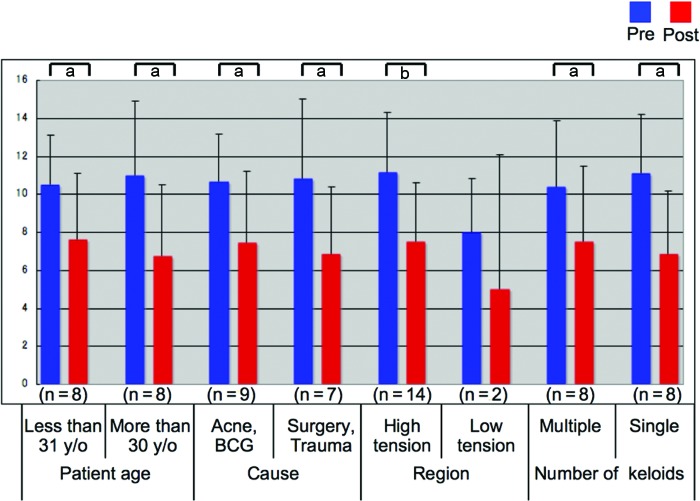
Average total scores of keloids before and after laser irradiation, after they had been divided into subgroups on the basis of patient age, cause, location, and number per patient. The keloids were divided on the basis of patient age (less or more than 31 years of age), cause (acne, bites, BCG vaccinations, trauma, or surgery), region (high tension sites such as the anterior chest wall vs low tension sites such as the face), and the number per patient (multiple or single). The average total scores of these subgroups before and after laser treatment were then compared by paired *t* test. Apart from the low-tension site scars, the keloids in all groups improved significantly after laser treatment (*P* at least <.05). *P < .05, † *P* < .01. y/o indicates years of age.

The keloids were then divided into subgroups according to patient age (less or more than 31 years of age), cause (acne, insect bites, BCG vaccinations, trauma, or surgery), location (high tension sites such as the anterior chest wall versus low tension sites such as the face), and number (multiple or single). The average total scores of the subgroups before and after laser treatment were then compared by using Mann-Whitney *U* tests. All subgroups apart from the low-tension site scar subgroup showed significant improvements in their average total score after laser treatment (*P* at least <.05; Fig 3).

**Figure 4:**
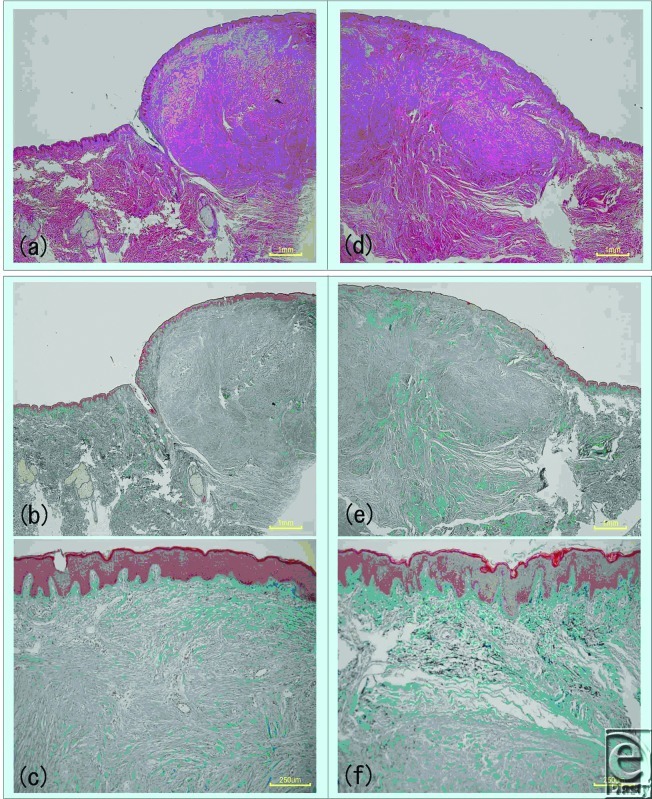
Changes in keloid tissue after laser irradiation, as observed by light microscopy. Half of the precordial keloid of a 26-year-old woman was irradiated with laser. Three days later, the unirradiated (*left*) and irradiated keloid halves (*right*) were excised and examined by hematoxylin and eosin staining (*top images*) and Elastica Masson-Goldner (*middle and bottom images*) staining. Structural changes were observed in the collagen of the irradiated tissues. Intense inflammatory cell infiltration was observed by hematoxylin and eosin staining.

**Figure 5:**
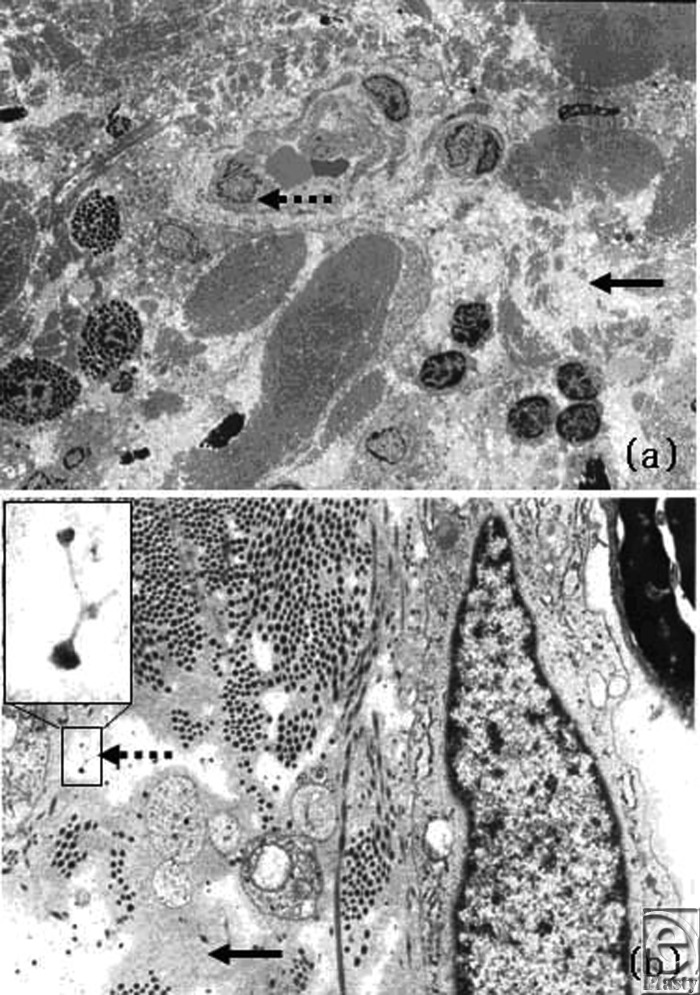
Changes in keloid tissue after laser irradiation, observed by electron microscopy. A change in the collagen fiber fascicles (*solid arrow*) was seen at × 2000 magnification, but changes in the vascular structure were not observed (*broken arrow*) (*above*). Lymphocyte aggregates were observed (*above*). Plasma protein leakage (*solid arrow*) and proteoglycan particles (*broken arrow and inset*) were observed (*below*).

Hematoxylin and eosin staining and Elastica Masson-Goldner staining of treated and untreated keloids revealed that laser treatment induced a structural change in the collagen of the tissue (Fig 4). This influence reached a depth of 0.5 to 1 mm. Electron microscopy revealed plasma protein leakage, proteoglycan particles, and a change in the collagen fiber fascicles (Fig 5). This is significant because there is considerable evidence that proteoglycans are bound to collagen fibrils and that anything that seriously disrupts the collagen meshwork entrapping proteoglycan aggregates will inevitably lead to the release of proteoglycans from the matrix. Changes in vascular endothelial cells and fibroblasts were not observed. Thus, this usage of the Nd:YAG laser does not appear to induce blood vessel coagulation. Rather, it seems to act via different mechanisms to reduce several scar symptoms.

**Figure 6:**
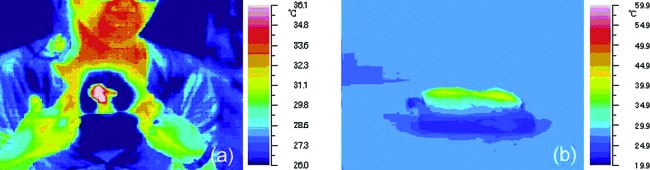
Heat-induced changes in a keloid scar and a raw pigskin, as determined by thermography. The keloid was irradiated with the laser and its temperature was measured when pain was induced. The average temperature that induced pain was 44.5°C (*left*). In raw pigskins, a rise of temperature was observed at a depth of about 1 mm under the epidermis (*right*).

Thermography of several scars during their laser treatment revealed that the skin surface temperatures were between 43°C to 46°C (an average temperature of 44.5°C) when the patients complained of heat sensation during a laser therapy session (Fig 6). Irradiation of raw porcine skins followed by thermography showed the temperature rise occurred at a skin depth of about 0.5 to 1 mm. This indicates that the laser will not reach the deep layer of scars if the scar has a thick collagen layer. Notably, it is known that temperatures above 54°C are required to thermally denature collagen fibers.^13^ Thus, the data obtained from this investigation suggests that noncontact mode Nd:YAG laser therapy does not appear to induce the thermal denaturation of collagen; rather, it induces collagen fiber fascicle decomposition.

## DISCUSSION

One of the most important effects of lasers in treating scars is that they generate heat, which initiates inflammation and in turn elevates vascular permeability, matrix metalloproteinase (MMP) production, and collagen fiber fascicle decomposition. Paquet et al^14^ have suggested that the increased vascular permeability and collagen fiber fascicle decomposition that is observed after laser irradiation is due to the release of MMPs. This notion is supported by Kuo et al,^34^ who found that PDL therapy of keloids stimulated the production of MMPs such as collagenase. This effect is considered to be more pronounced with the Nd:YAG laser because it reaches greater depths than a PDL. Clinical findings from 2 cases (cases 1 and 2) support this theory since neither case exhibited scarring in the keloidal area after it had been restored by Nd:YAG laser therapy. Because other short wavelength lasers do not appear to significantly induce MMP production, it may be that different lasers have different effects on scars.

Supporting this is that electron microscopic analysis of keloid scars after one half of each scar had been treated with the noncontact mode Nd:YAG laser showed that there was no immediate change in the vascular endothelial cells after therapy. Clear intravascular blood coagulation or vascular obstruction was also not observed. In contrast, Paquet et al^16^ have suggested that PDL improves keloids or HSs by inducing capillary destruction, which generates hypoxemia and in turn alters the local collagen production. Dierickx et al^17^ have also attributed the therapeutic effect of PDLs to hypoxemia resulting from laser-induced heat and vascular injury.

Electron microscopy also revealed the disruption of collagen bundles and proteoglycan particles in the interstice. A *proteoglycan* is defined as a protein that bears one or more glycosaminoglycan chains. There is considerable evidence that proteoglycans are bound to collagen fibrils and that anything that seriously disrupts the collagen meshwork entrapping proteoglycan aggregates will inevitably lead to the release of proteoglycans from the matrix.^18^^,^^19^ Thus, it appears that there were proteoglycan particles in the interstice because the therapy had disrupted the collagen bundles.

Electron microscopy also detected extravascular leakage of plasma protein, which suggests that Nd:YAG laser treatment may have increased vascular permeability. Histology showed that noncontact mode Nd:YAG laser therapy induced structural changes in the collagen-bearing tissue at a depth of 500 to 1000 μm. In contrast, 585 nm PDL is known to change the heat of target vessels that are 71.6 to 100.3 μm deep.^13^ In addition, the irradiation of raw pigskins followed by thermography revealed that noncontact mode Nd:YAG also induced heat at a depth of about 500 to 1000 μm. The papillary and reticular dermis is located approximately 100 to 500 μm below the skin surface. These observations suggest that the thermal heat of the noncontact mode Nd:YAG laser reaches the middle layer of the dermis, where keloids develop. Nd:YAG laser energy is weakly absorbed by melanin and water as well as longer wavelength, laser beam penetrates deep into the skin. This finding accounts for the histological change in the collagen fiber fascicles.

In a previous study, histology of a port wine stain that had been treated with contact-mode Nd:YAG laser at 130 J/cm^2^, 6 ms, 5 mm spot revealed that the deepest vessel damage was 2 mm from the dermoepidermal junction.^20^ Together with our own observations here, this suggests that the thermal effects and vascular damage induced by noncontact mode Nd:YAG laser occur much deeper than those generated by PDL but less deeply than Nd:YAG in contact mode. In this study, we used Nd:YAG in the noncontact mode to treat scars without inducing pain. However, should we be able to treat scars painlessly by using the Nd:YAG laser, our results suggest that the contact mode will have better clinical outcomes than the noncontact mode in the treatment of keloids and HSs.

## CONCLUSIONS

This clinical evaluation found that noncontact mode Nd:YAG laser treatment is highly effective for both keloids and HSs, regardless of patient age, the origin and multiplicity of scarring, the location of the scar(s), or the tension on the scar. However, the ability of noncontact mode Nd:YAG laser therapy to treat multiple active big scars is limited because its efficacy decreases with the thickness of the scar. Moreover, such scars would require a large number of laser irradiations, which would be too time-consuming and costly for most patients. Thus, this laser treatment modality is particularly helpful for keloids and HSs that have just appeared and/or have remained small and thin.

In the present study, the only keloid subgroup that did not exhibit significant changes in average total scores after treatment was the low-tension site scar group. However, the sample size of this group was small. Thus, the effectiveness of noncontact mode Nd:YAG laser treatment for low tension sites remains unknown. This question should be addressed with a greater number of subjects. In addition, a randomized split scar study is needed to show whether the noncontact and contact modes of Nd:YAG laser alone or in combination differ in efficacy. A similar study examining the efficacy of combination therapy with a dye laser and Nd:YAG would also be of interest.

### [Case 1, No. 2]

**Figure 7:**
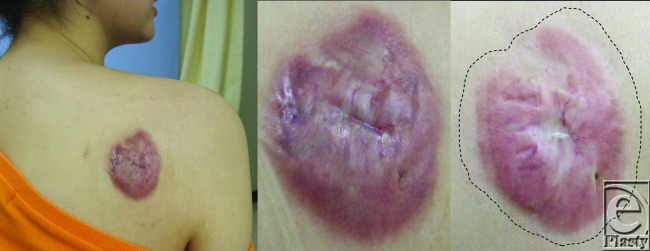
Case 1.

The patient was a 26-year-old women with a keloid on the right shoulder that was the result of acne. Although the lesion had been excised and treated with electron-beam irradiation 4 years previously, it subsequently returned. The patient was then treated 5 times with a pulsed dye-laser and 3 times by local steroid injection at a different clinic. However, the lesion continued to grow and the patient visited our hospital. The patient had a menstrual disorder as an adverse reaction to the steroid injections, and surgical treatment was not an option. Consequently, the patient agreed to Nd:YAG laser therapy (Fig 7). All parameters were measured after a total of 22 treatments over 10 months. Significantly, some of the keloidal areas were restored to nearly normal skin.

### [Case 2, No. 5]

**Figure 8:**
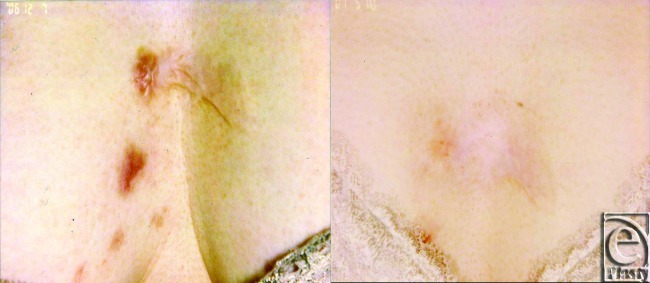
Case 2.

The patient was a 64-year-old woman with a keloid caused by an insect bite on the chest. After 7 Nd:YAG laser treatments over 5 months, the keloid disappeared (Fig 8). The itchiness and pain were also reduced. At present (more than 6 months after the last treatment), the keloid has not reappeared.

### [Case 3, No. 17]

**Figure 9:**
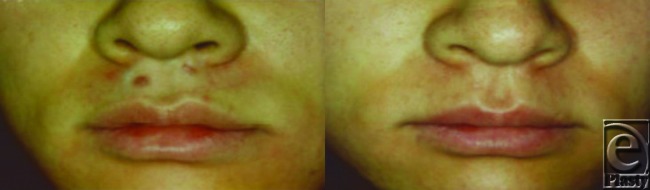
Case 3.

This patient was an X-year-old woman with postburn hypertrophic scars caused by the removal of hair on the upper lip by laser (Fig 9). After 5 Nd:YAG laser treatments, the erythema and elevation of the lesions were reduced.

## Figures and Tables

**Table:**